# A system dynamics approach for understanding community resilience to disaster risk

**DOI:** 10.4102/jamba.v13i1.1037

**Published:** 2021-06-14

**Authors:** Onyekachi J. Onyeagoziri, Corrinne Shaw, Tom Ryan

**Affiliations:** 1 Department of Mechanical Engineering, Faculty of Engineering and the Built Environment, University of Cape Town, Cape Town, South Africa; 2 Graduate School of Business, University of Cape Town, Cape Town, South Africa

**Keywords:** disaster risk reduction, community resilience, grounded theory, system dynamics, informal settlements

## Abstract

The Western Cape is a dynamic province that is disaster-prone, particularly the vulnerable urban communities in and around its environs. Such communities are more vulnerable to wildfire, flooding, pandemic, natural and human-made hazards because of poverty and, consequently, poor living conditions such as overcrowding and non-understanding of community resilience. The inability of these communities to understand community resilience and withstand adversities affects the sustainability of initiatives to develop them. This study aims to identify the mechanisms influencing the level of understanding of community resilience in a vulnerable community and to contribute to the understanding of community resilience to disaster risk. Fieldwork was conducted in an informal settlement in South Africa. The research study was conducted in two cycles of data collection and analysis. Data in the form of observation notes, document analysis and interviews were analysed using grounded-theory principles. Ten inter-related variables or mechanisms emerged from the analysis. The theoretical model consists of four reinforcing (R) feedback loops (R1, R2, R3 and R4), respectively, which explain how the understanding of community resilience in the informal settlement maps on to the relative achievement systems archetype. Negative reinforcing behaviour would explain the lack of understanding of community resilience, while positive reinforcing behaviour indicates how an understanding of community resilience develops. In addition, the variable with the leverage to improve the mechanisms influencing the understanding of community resilience was found to be the ‘level of public education and awareness’. The theory of how these variables behave in context was represented as a qualitative system dynamics model.

## Introduction

Several reports have declared Cape Town as the most disaster-prone city in South Africa, with the highest rate of disaster-related deaths, which mostly occur in informal settlements (Walls et al. 2017). Globally, one of the leading causes of death because of disasters is a particular kind of disaster called fire disaster and this accounts for 96% of all fatalities or burn deaths, totalling 300 000 deaths per year in low- to middle-income countries (LMICs) (Mock et al. 2009). This type of death is the fourth most significant injury caused by accident globally (Twigg et al. 2017).

Disasters could steadily jeopardise South Africa’s populations, economy and the sustainable community development of the nation (Hyogo Framework for Action [HFA] 2012–2015). For example, there is an unequalled load of injury, disadvantage and disability emerging from disaster in South Africa (Western Cape Strategic Framework for Fire and Burn Injury Prevention 2015). If plans for appropriate risk management are not prepared before the disasters, it could lead to consequences that might include economic losses or deaths (Isa, Sugiyanto & Susilowati 2018). Therefore, it is essential to carry out mitigation and adaptation strategies before disasters to reduce the consequences and effects, thereby helping to reduce disaster risk (DR) (Isa et al. 2018).

There have been various approaches to disaster risk reduction (DRR). Gaillard and Mercer (2013) referred to top-down approaches and contrasted these with bottom-up approaches. In top-down approaches, scientific formal knowledge from people and groups outside of the ‘at-risk’ communities design and initiate interventions on behalf of the community. In bottom-up approaches, local knowledge is utilised by communities and marginalised groups to manage DR. Practitioners in the field have been calling for inclusion of those affected by disasters in the development of policy and actions in DRR (Gaillard 2010; Heijmans 2009; Pelling 2007).

The bottom-up approach values local knowledge and recognises that communities could play a role in managing DR. An integrative approach that combines bottom-up and top-down approaches is argued for by Gaillard and Mercer (2013). Furthermore, Disaster Management Training and Education Centre practitioners at the University of the Free State (UFS) in South Africa introduced a theory of DRR through environmental design. The purpose of this initiative was to reduce DR by using the built environment to impact elements that add to DR as well as to reduce the general DR in urban environments.

The extent to which societies and citizens mitigate the risks and consequences of disasters is referred to as resilience (Paton & Johnston 2017). Ardalan and Paton (2015) used the idea of *community resilience* to think about challenges of DR and have asserted that it has become the basis of DRR and response. While numerous research efforts like Bergstrand et al. (2015) have assessed the relationship between DR and community resilience (CR) and how to improve resilience in many places, challenges still remain in replicating these results across contexts. Isa et al. (2018) explained that even in the absence of mitigation and adaptation mechanisms, the community should be able to decrease the impacts of disaster through resilience because community efforts and empowerments against disasters could produce a high level of CR.

In this study, DR in the Western Cape province is viewed as a complex adaptive system. This view challenges simple cause-and-effect assumptions and recognises that components in a system are connected and interact in ways that cannot be predicted. This study therefore aims to identify the mechanisms influencing the level of understanding of CR and how to intervene with respect to them for the purpose of improving resilience and reducing DR in the vulnerable community. To achieve this purpose, this research study focuses on the case of the Phola Park community in the Western Cape province to construct a theoretical model grounded in the empirical actualities of those involved in DRR in the Phola Park, and intends to answer the following research questions:

- What is the underlying mechanism(s) influencing the level of understanding of CR in a vulnerable community?
- What is the determinant(s) for improving the mechanism(s)?

This site was chosen as a case study for this research because at the time of this study it was an informal settlement that had experienced disasters and needed some level of understanding of CR. To address the complex problems relating to the challenges of DR and the need for understanding CR in the Western Cape, a system dynamics (SD) approach was used in conjunction with appropriate modelling techniques to identify and understand the variables in a community and how they interact when faced with possible disasters. System dynamics modelling has been employed for development planning and support-related decision-making. Brent, Simelane and Clifford-Holmes (2018) noted that this is achieved by simplifying reality in such a way that it can be employed to provide exploratory decision support.

The benefits of understanding the mechanisms contributing to resilience found in vulnerable communities include: (1) possible cost savings on disaster recovery or emergency relief and (2) improvements in CR and disaster response. Studies have shown that tackling vulnerability using resilience development is cheaper than emergency relief (Venton et al. 2012).

### Introduction to system dynamics approach and Vensim software

System dynamics, a particular system thinking approach, was used in this study to achieve the desired purpose of the study. System dynamics is a computer simulation technique which was firstly introduced at the Massachusetts Institute of Technology in 1950 by Jay W. Forrester. This approach has been applied extensively in the field of finance, healthcare, economics, environmental research, biology and information technology since the 1960s (Sapiri et al. 2017), and in 2020, it was applied in the field of CR and DRR (Onyeagoziri 2020). The narration of SD as an approach applied to feedback control and non-linear system in sciences and engineering, which was prolonged to non-professional fields started since the 1950s, and the goal was to decide how the failures or successes found in an industry can be decided by science and engineering (Sapiri et al. 2017).

System dynamics has a main goal to understand how the component in a system interacts with each other. The interactions in the components of a system are done through the feedback loops, which means that a change or increase in a component affects the other components (Sapiri et al. 2017), and a decrease in a component affects the other components.

The research problem is analysed as a complete or comprehensive ‘system’ in the SD modelling. Therefore, according to Sapiri et al (2017:1), ‘[*a*] system is defined as a collection of elements that interact with each other continually’. A system in this study is not a physical objective entity, such as a nuclear power station which would be considered a system, the understanding of a system is as a conceptual idea for viewing a problem situation based on Checkland’s views (Onyeagoziri 2020).

The feedback loop that comes in the form of the causal loop diagrams (CLDs) derived from a software called the Vensim (Sapiri et al. 2017):

[*E*]xplains the behaviour of a system by showing a group of nodes that are interconnected by arrows and the feedback loops created by the connections, the nodes represent elements in the real world that have been identified as the key variables in the system, and the arrows then show how a variable affects another variable. (p. 25)

The Vensim software mentioned above was established by ‘Ventana System’ when they were determined to put a stop to the reconstruction of old software and decided to make simulation languages of their own, which is now referred to as ‘Vensim’ (Sapiri et al. 2017).

### Defining resilience to disasters

Holling (1973:14) firstly used the term ‘resilience’ to describe a ‘measure of the perseverance of systems and their capability to absorb change and disturbances and still maintain the same relationships between populations or state variables’. Klein, Nicholls and Thomalla (2003) and Manyena (2009) noted that there is no single definition of resilience. It is a concept that Kaplan (2003) describes as a term that is commonly challenged by hidden complexities, contradictions and vagueness. This is because different levels of understanding must be considered when trying to understand resilience in communities.

According to Cutter et al. (2008), resilience is defined as the capacity of a social system to recover from disasters and having characteristic states that enable the system to absorb shocks and cope with post-disaster events, adaptive procedures that promote the capacity of the social system to withstand threats. Adger et al. (2005), Folke (2006) and Klein et al. (2003) claim that resilience also does not only involve a system’s capacity to fall back to the state or several states existing prior to disturbance, but to promote the state by learning and adaptation processes. This is the approach to resilience that is considered in this study. Resilience is also defined as a system’s ability to absorb shocks and restructure into a complete performing system (Mitchell & Harris 2012; Walker et al. 2004).

Cutter et al. (2008) argued that there is substantial research interest in the measurement and meaning of resilience from several research approaches, including those studies focused on disasters and global change environments. Although there might be an identification of the risks in many communities, hazard reduction and vulnerability are mostly not of significant interest until after there is a disaster occurrence, because the community members seem to have other issues that are a priority to them (Cutter et al. 2008). In addition, government-elected officials might not want to linger over the vulnerability of their communities, as they feel that it may affect economic investment and growth.

After looking at the available definitions of resilience, the researcher has chosen a definition of resilience that is appropriate for this research project. In this research study, resilience is therefore defined as a positive turnaround, outcome, or adaptation of a competent individual or community exposed to risk, difficulties, significant challenges or adversities. This is because people need to know and understand resilience to be able to build it within themselves or their communities in the context of withstanding DR (Cutter et al. 2008; Lew et al. 2016).

### Community resilience

Community resilience has similarities with the individual resilience because a community is made up of individuals. In a report given by the Community and Regional Resilience Institute (CARRI), there were 25 CR definitions given by several researchers (Eachus 2014).

The CARRI (2013) defined CR as the ability of communities to forecast disaster, reduce the impact of disaster and can quickly recover through growth, evolution, survival and adaptability during shocks, adversity and difficulties. Joerin et al. (2012) also defined CR as the ability of communities to absorb, cope and rapidly recover during disasters.

Community resilience has contributing elements or factors. Several researchers have developed factors that contribute to CR. For example, Joerin et al. (2014) have contributed to CR by producing the ‘Climate Disaster Resilience Index’ (CDRI), which focuses on 5 dimensions (which are the institutional, physical, economic, natural and social dimensions), 25 parameters and 125 variables, which considers the capability of individuals and institutions to recover from climate-related disasters in India.

Also, in acknowledging the contributions of current models and their restrictions around resilience and vulnerability, there is a model called the ‘DROP’ model for disaster resilience, which was designed to show the theoretically grounded connection between vulnerability and resilience; and this model can be applied to tackle real challenges in real places (Cutter et al. 2008:602).

Community resilience is seen as a dynamic and fundamental characteristic of DRR, which means that resilience should be a part of the community’s life cycle (CARRY 2013). This could enable the community to adapt and recover from disasters. Therefore, understanding CR and the importance thereof is essential for the vulnerable communities or the individuals in the community because the community consists of individuals and CR could be determined by individuals (Hegney et al. 2007). However, this is driven by regulations, customs, cultures, manners and common practices. Eachus (2014) stated that if some individuals in the communities are non-resilient, that it is still plausible for the communities to be resilient and this is considered in this study.

### Disaster risk reduction

Disaster risk reduction is the concept of practices used to reduce DR by systematically carrying out a detailed analysis and reduction of the factors causing the disasters (United Nations Office for Disaster Reduction [UNISDR] & [WMO] 2012). Also, because of several definitions of disaster, from several sources, which have been used for several purposes, it is important to indicate what definition is required and related to this article. Therefore, for this article, ‘disaster is a social scientific concept that refers to a particular class of phenomena whose specification rests in theory-based thinking’ (Ronald 2017:2). Much can still be performed to protect informal settlements in South Africa against disasters. This has been shown in countries like Cameroun and Malawi, where high-tech community-managed system-based scientific approaches were applied to reduce the negative effects of disasters (Twigg 2004). However, it is not yet recorded that these approaches could successfully be used in the Western Cape or South Africa for DRR.

Disaster risk reduction is linked to sustainable development, which involves regulation and training around key disciplines that include disaster management, disaster mitigation and disaster preparedness. Everyone’s involvement is necessary to develop and sustain these activities and disciplines in order to reduce DR (UNISDR & WMO 2012). Therefore, to enable successful community sustainable development, DRR should involve all the key societal stakeholders: the government, the private sector and disaster management professionals. This is also in agreement with this research study, as no one person can build and sustain resilience against disasters in a community or nation.

The gravity of a disaster depends on the extent of the effect of hazards on the communities, as the level of the effects depends on the choices made by the people for their lives and communities (UNISDR & WMO 2012). These choices are related to how food is grown, how homes are constructed, the kind of government in power, the policies enacted, the support of financial systems and education, that is, what people are taught in schools (UNISDR & WMO 2012). The decisions or choices made could be what makes the communities either more vulnerable to DR or more resilient to DR. Therefore, understanding and development of CR should be based on better choices or decisions made by governments, communities and all the people involved.

### Communities and disaster risk reduction

In 2010, shelter cluster indicated that a community or an environment has an important part to play in the DRR and the effect of disaster events, because proper ecosystem management can mitigate the risk of hazards such as landslides, flooding and storm surges. Guion, Scammon and Borders (2007) also stated that:

[*D*]isasters were considered to be an act of God which are beyond human control, but today there are widespread agreements that although disasters cannot be controlled, their effects can be managed. (p. 20)

For more than 50 years, the four-phase traditional model of disaster management – namely, mitigation, preparedness, response and recovery – has been utilised and has informed several research studies on DRR (Mileti 1999).

This four-phase traditional model could work better when understanding of CR is part of the model. This is because preparedness, response and recovery without understanding CR in the informal settlements might lead to the recurrence of disasters (Cutter et al. 2008). Therefore, to attempt DRR, the communities have a very vital role to play for a proper and thriving community sustainable development.

## Research methods and design

In the next section, the context for the study is described and the methodological aspects of the research design, including SD modelling, are explained. Then, a detailed explanation of the theory building process is provided to demonstrate how isomorphic analogy is used to support the development of a grounded theory.

### Context for the study

This study was conducted in the Phola Park community of the Western Cape province in South Africa. Phola Park is an informal settlement located along an arterial road in an area designated for development and is similar to other informal settlements in Cape Town (Haferburg 2002).

The Phola Park community is exposed to flood, wildfire and other DRs. This area consists totally of shacks (houses built by hand with available materials) that are frequently prone to disasters because of inadequate drainage system, the lack of housing and general facilities such as toilets and water taps and overcrowding.

### Data collection and analysis

This study takes an inductive, grounded theory approach for understanding CR to DR. This article aims to identify the mechanisms influencing the level of understanding of CR and to use these as a basis for recommendations for reducing DR.

Data were gathered through observations and interviews. Participatory observations were performed over 3 months with weekly site visits as a process of gathering data through involvement (widespread community participation) and exposure to day-to-day activities in the informal settlement. Community activities, such as meetings, were also attended by the researcher. A total of 10 members of the community who had personal experiences of DR participated in a focus group.

Semi-structured interviews were conducted with 30 people, mainly youths from the community and the community leader. These people were identified as the most trusted and ardent community members in Phola Park at that time. They were each interviewed for 20–30 min. The choices made with respect to sampling, settings or individuals to be observed and interviewed were informed by the aim to provide credible data rather than representativeness or the capacity to generalise (Nastasi, Moore & Varjas 2004). Data were analysed using grounded theory principles.

The analysis was conducted simultaneously with the data collection, focusing on the interviews and observations concurrently. Categorising strategies (open coding, axial coding, selective coding and thematic analysis) and connecting strategies (including narrative analysis and individual case studies, memo and displays) were applied. The summary of the research process from the data collection stage to the analysis or the grounded theory process is depicted in Online Appendix 1.

Access to the informal settlement was facilitated by a non-governmental organisation that was working on a project with the Phola Park community.

### System dynamics application

System dynamics is an approach for modelling complex systems. A context or problem can be conceptualised as a system with interacting variables and SD provides techniques for modelling the behaviour of such a system, including identifying the most impactful elements of the system (Brent et al. 2018). System dynamics models use CLDs to represent causal relationships between variables that make up a system (Forrester 1973, 1987, 1991), and they have been used to indicate complex relationships and feedback results within many sectors (Vennix 1999). According to Sterman (1991), SD also provides the policymakers with tools for identifying solutions for complex challenges.

### Overview of the theory-building process

The theory-building process involves the identification of concepts from the grounded theory analytical process. These concepts informed a review of literature to extend on the empirical definitions and contribute to the grounded theory. Then, the theory was further developed using an analogy process as developed by Beer (1994).

**FIGURE 1:**
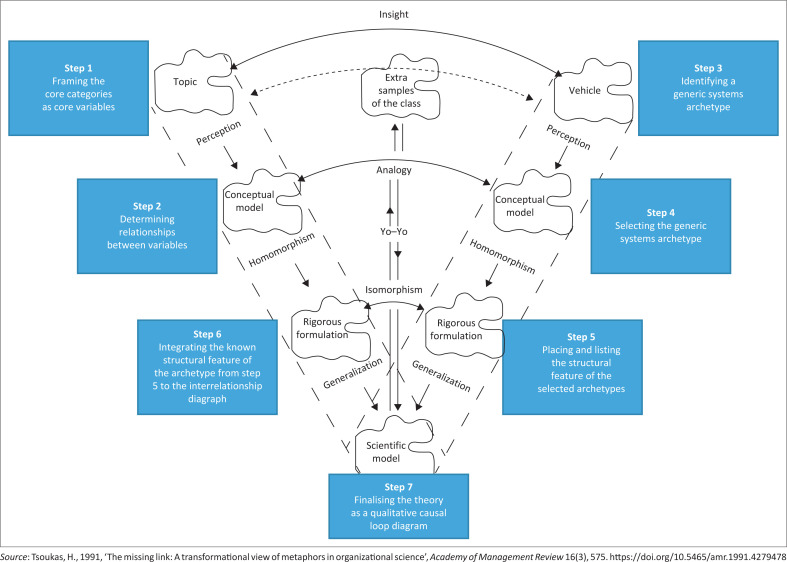
The process of building a theoretical model: ‘A transformational view of metaphors in organisation science’.

**FIGURE 2:**
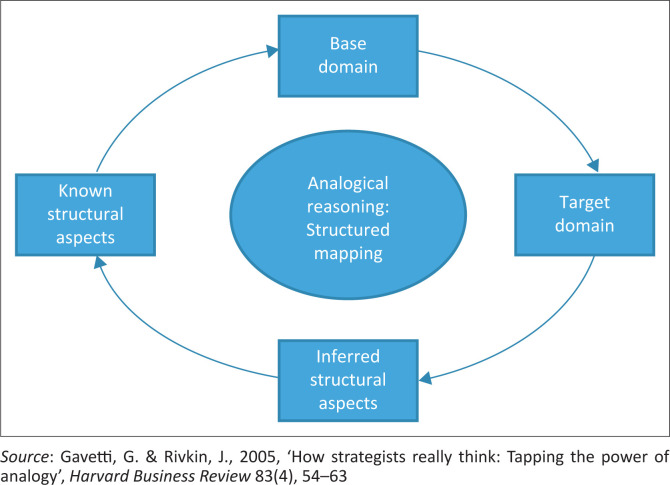
A representation of the structured mapping procedures, explaining the analogical reasoning.

In this study, theory-building involves analogy and isomorphism steps that are illustrated in Figure 1. Here, the isomorphism and analogical steps are used for the rigorous construction of a theoretical model that provides an answer to the research questions. Gavetti and Rivkin (2005) said that an analogy is the identification of an item called the base domain, which is not strictly the same as the target domain. The structure mapping process (Figure 2) allows for the construction of a new theory during the result building process, depending on the conclusions reached, because the analogical reasoning is a complex segment of the result building procedure.

**FIGURE 3:**
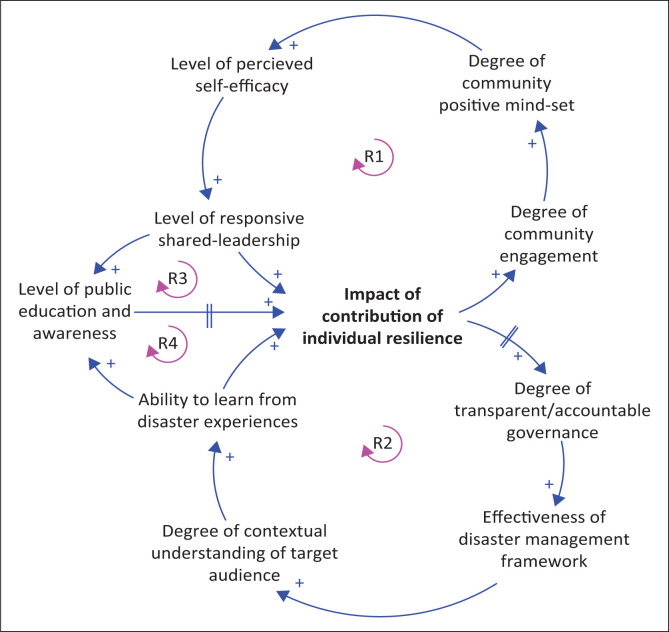
The integrated theoretical model of understanding of community resilience based on a community vulnerable to disaster risk.

Kieras and Bovair (1984) proposed two approaches to analogical reasoning. The first one allows for a better insight on the operation of two means, and an inbuilt understanding of how the means functions allow for the reasoning of how similar means operate or to understand how a system works. The second type is an insight that is used for reasoning, and it is already inbuilt to dictate a solution to a problem; this is the domain insight (Kieras & Bovair 1984). Leboea (2017:79) stated that ‘in the theory-building process, the base domain metamorphoses into the Wolstenholme generic archetypes (see Online Appendix 2), and the target domain discovered becomes the inter-relationship diagram and the variables’ (see Figure 3).

#### Step 1: Framing the core categories as core variables

Concept analysis was performed on the 10-core categories generated in the study, which provided more insights into the core categories. In this step, the analysis of the data derived from the study was used to choose a variable for the categories within the context of the study and paying attention to its importance.

#### Step 2: Determining relationships between variables

The relationships between variables were tested using an interrelationship diagraph, a tool for exploring for evidence of links or influences between each of the variables identified.

#### Step 3: Identifying a generic systems archetype

In step 3, a generic archetype was chosen from the Wolstenholme generic archetypes and Braun’s archetypes (Braun 2002; Wolstenholme 2003), depending on the details and understanding derived from them. Further explanations of the archetype are represented in the next section.

#### Step 4: Selecting the Wolstenholme systems archetype

Selection of appropriate Wolstenholme systems archetype as known theories of systems behaviour that maps to the empirical findings.

#### Step 5: Placing and listing the structural feature of the selected archetypes

This step involves the identifying feedback in the causal loops, how they affect each other and the outside environments. The process allows for the documentation of the identified known structural features of the selected archetypes.

#### Step 6: Integrating the known structural feature of the archetype from step 5 to the interrelationship diagraph

This is where the CLD (SD model) is generated, which means that the new findings and understanding derived from the CLD of the research work are explained here. This includes inferring the variables from the CLD into an essential segment of the selected systems archetype in a way that makes it sensible.

#### Step 7: Finalising the theory as a qualitative causal loop diagram

As soon as the structural feature of the selected systems archetype is inferred into the interrelationship diagraph, the finalised model can then be constituted as a CLD that corresponds precisely to the concern variable or research problems.

### Ethical considerations

Participation in the study was voluntary; thus, no benefits or cost was determined or given. However, there were refreshments provided for those who needed them. Interviews were also conducted at a time and place convenient for the interviewee and telephonic interviews were conducted if the participants were not available to meet up. Before the interview, the participants were informed that they could stop at any time and consent form was signed by the participants who would allow for the use of audio recording.

This research study received ethics approval from University of Cape Town’s ethics committee: ONYJOH001.

## Results and discussions

In this section, the findings of the application of the research process are presented. These findings are described below as two cycles of theory building: (1) cycle 1 draws on data from a systematic literature review and (2) cycle 2 draws on the empirical data.

### Cycle 1

A total of *83 propositions* and *30 categories* that were related to the concern variable of development of CR were generated. The categories were later condensed to *seven-core categories* through reduction sampling, and a CLD was generated as the research result for research cycle 1 (see Online Appendix 3).

Research cycle 1 served to focus on the study and provided a foundation for the theory building in cycle 2. The role of literature in cycle 1 was as a source of data rather than to provide a conceptual framework.

### Cycle 2

Research cycle 2 was built on cycle 1. In this cycle, the interview transcripts from individuals in the case study and journal entries from observations served as data. The cycle 2 concern variable or object of interest was centred on ‘level of understanding of community resilience’. From the data gathered in the research cycle 2, a total of *192 propositions* and *60 categories* related to the concern variable and research topic were generated. The categories were later reduced to *10 core categories* (see Table 1). At this stage, the use of *generic archetypes* was also used to develop the CLD as the research result for this article (see Online Appendix 2).

**TABLE 1:** Core categories framed as core variables (renamed variables).

Core categories	Core variables
Contribution of individual resilience to community resilience	Impact of contribution of individual resilience
Community engagement (continuous, cohesion and inclusive)	Degree of community engagement
Positive mindset and readiness of the communities	Effect of community positive mindset
Public education and public awareness on community resilience	Level of public education and awareness
Ability to learn from disaster experience for community resilience	Ability to learn from disaster experience
Transparency and accountability governance of disaster team	Degree of transparent or accountable governance
Perceived self-efficacy to community resilience	Level of perceived self-efficacy
Contextual understanding of disaster risk and target audience	Degree of contextual understanding of target audience
Responsive shared leadership	Level of responsive shared leadership
Disaster management framework on community resilience	Effectiveness of disaster management framework

The research milestone, which is documented separately in (Online Appendix 1), shows a brief synopsis on the 60 categories generated.

The understanding gained from the cycle 2 is that in areas exposed to DR, if the resilience from the people living in these areas is understood and contributed to the community, it could contribute to improved CR, thereby attempting to reduce DR.

### Applying the result

The structural mapping explaining the analogical reasoning used in the theory-building process application is presented in Figure 2, which will allow for the construction of the new theory, depending on the conclusions reached in the result building process. The structure mapping is described as a theory used to interpret analogical reasoning (Gavetti & Rivkin 2005).

The section below illustrates the application of the process of theory building, which was used to achieve the result of the research study. The intention for this is not for repetition, but to systematically illustrate the processes.

#### Step 1: Framing the core categories as core variables

Through the application of grounded theory principles, 10 core categories where developed. The 10 core categories developed, and the variables re-named dynamically, are shown in Table 1.

#### Step 2: Inter-relationship diagraph of core variables

The interrelationship diagraph explains the core variables. It was developed by using the variables chosen from the core categories and mapping the direct relationships and indirect relationships (see Online Appendix 4).

#### Step 3: Identifying a Wolstenholme generic systems archetype

The relative achievement archetype is when achievement is realised at the detriment of another sector (Wolstenholme 2003). Wolstenholme (2003:7–26) indicates that this type of archetypes is made up of a reinforcing loop that intends to attain a comparative advantage from inventiveness (see Online Appendix 2). The concern variable is the level of understanding of CR, which means that the research wants to increase something. The framework of Braun has shown the path that is most important to the concern variable of the research study.

#### Step 4: Initiating the identified Wolstenholme systems archetype and selecting a Braun archetype

Wolstenholme (2003), stated that:

[*T*]he key to identifying solution archetypes lies in understanding both the magnitude of the delay and the nature of the organisational boundary present, and the solutions required by system actors when instigating a new action should attempt to remove or make transparent the organisational boundary masking the side effect. (p. 5 [see Online Appendix 5])

#### Step 5: Placing and listing the structural feature of the selected archetypes

This process involves the interaction of the causal loop feedback and the environment. This is where the structural features of the selected archetypes are recorded. This shows the structural archetype selected for placing and listing (see Online Appendix 2).

Success to successful archetype structure explains that growth in a system leads to a decrease of the next system. Anderson and Kim (2011) asserted that:

In success to the successful situation, two or more individuals, groups, projects, initiatives, systems are vying for a limited pool of space to gain success, when one of them starts to become more successful (or is historically already more successful) than the other, it tends to garner more spaces, thereby increasing the likelihood of continued success. (p. 87)

#### Step 6: Inferring the known structural feature of the archetype from step 5 to the inter-relationship diagram (inferring the core variables)

This is a process where the variables derived are inferred appropriately into the selected segments of the systems archetype in a way that their relationships are in line with the other variables inferred into the systems archetype. This should also speak to the concern variable in the understanding of the CLD.

Studies have indicated that most CLDs approximate generic CLDs (Kamprath 2014; Kiani et al. 2009; Anderson & Kim 2011; Ríosb 2006). What this means is that the CLD developed in this study would most likely be one of the generic archetypes. Online Appendix 2 shows the inferring of the known structural feature of the archetype to the causal diagram. The variables were inferred to the structure to match it appropriately.

Archetypes are necessary for getting knowledge into the ‘nature’ of the underlying issues and for giving an initial foundation upon which a model can be further developed and established. The aim is to achieve something at the maximum level or lowest level that can be illustrated by a reinforcing loop. The archetype chosen is to control something, and that led to the decision of the loop applicable to the research. This means that the respective generic CLD was selected because it applies to the research focus.

The research variables were looked at, and the researcher considered how they would fit into the generic CLD. Anderson and Kim (2011:91) asserted that [*t*]he key to diagramming success to the successful diagram is identifying the central variable involving choice and allocation of resources’.

The unique design of behaviour over time for the success of the successful archetype is more of a diverging curve, where one goes up and the other goes down. This means that if the researcher represented resources A as CR and resources B as DR, it means that the success or increase in the level of CR will lead to the decrease of the DR. It also means that the CR is saying to the DR that the increase leads to your decrease. This is illustrated by the hypothesis in Online Appendix 6.

#### Step 7: Finalising the model (the theoretical model for this research study)

The theoretical model above explains how the behaviour of the relative achievement archetype relates to the level of understanding of CR. This explains the mechanisms that influence the level of understanding of CR in the Western Cape.

Relocated themes from the relative achievement model, which was used to correspond with the variables from the inter-relationship diagraph, are illustrated in Online Appendix 2, which recognise the themes in the success of successful CLD. These themes were assessed, and insights were given considering their effects to the other themes in the CLD. Interchangeable loops were found through the inter-relationship diagraph derived from the research process, and more insights obtained from the review of the literature were used to equal the variables of the CLD through interchangeable attributes to the generic archetype.

The equality might not be in perfect state, but the loop of the relationship remains in shape (see Online Appendix 2). This directed the researcher to go beyond the simple archetypes by embedding the variables inferred in the archetype in a system that is more complex or selecting the archetype in more depth and range of applications to actualise the theoretical model or research result required for this article (see Figure 3).

The compatible variables were inferred into the relative achievement archetype to develop the CLD, which is related to the concern variable of understanding of CR in the Western Cape.

The theoretical model provides an insight into how similar concepts and core variables interact with each other to develop or influence the understanding of CR. The variables are also the identified mechanisms influencing the level of resilience of the vulnerable community.

To further explain the model, a description is provided below for the four reinforcing feedback loops.

### Theoretical explanation of the model

The theoretical model is made up of individual core variables interacting together as reinforcing feedback loops, which indicates the causal relationships existing in the CR model. The nature of the variables makes them difficult to measure quantitatively as they require access to the experiences of people. The dynamics of these relationships are explained next.

The four feedback loops in the model (Figure 3) explain the reciprocal relationship between public education and awareness, and understanding of CR through interlinking variables and loops. Reinforcing loop 1 (R1) and reinforcing loop 2 (R2) both demonstrate how aspects of agency in individuals can contribute to the level of understanding of CR. R1 explains the attributes of individuals, such as community engagements, positive mindsets, self-efficacy, responsive shared leadership and individual resilience that contribute to a positive outlook, while R2 represents skills in relation to transparent governance, effective disaster management framework, understanding the target audience and learning from disaster experiences. This combination of attributes and skills influences the degree to which individuals understand resilience in the community. Public education and awareness in this sense represents the ability to improve these mechanisms or attributes. This ability therefore can contribute to the contribution that the individual makes to the understanding of resilience of the community.

In the systemic structure of the CLD (model), there is a need to improve these mechanisms to influence CR; this explains the reinforcing loop 3 (R3) and reinforcing loop 4 (R4) on the model (see Figure 3).

As noticed, all these variables were developed systematically and are to influence the ‘level of understanding of community resilience’, which is the concern variable of this research study. Therefore, the question of the mechanisms influencing the level of understanding of CR in the vulnerable community was answered.

The answer shows that the mechanisms that influence the level of understanding of CR are the nine variables in R1 and R2. The variable ‘level of public education and public awareness’ on CR is the determinant one to improve the core mechanisms for better understanding of CR (see Figure 3).

## Validity

There is a possibility that the theoretical model can be termed adequate regarding the real-world situations. There is also a possibility that the model was developed because of what would like to be seen and not what is happening, these possibilities were handled.

For the enrichment and maintenance of the validity, the research design was iterative. To enrich the validity of the study, the researcher took part in defining the conceptual framework from the start of the research study to provide the trustworthiness criteria of credibility and confirmability, increasing the data collection reliability, using theoretical sampling to check the upcoming ideas, using the literature review and used the different coding processes to get trustworthiness criteria of dependability.

For the transferability of the derived theory, diverse sources of data were used for the enrichment of the validity of the research study. The analogical stance was also used to apply the systems archetype to the related concepts allowing for the alignment of accepted models used to develop the theoretical model, thereby increasing the theoretical validity.

## Limitations and recommendations

The theory generated is based on data collected in the Western Cape province, that is, in a single context. Therefore, it can serve as a hypothesis that could allow for further study to explore the transferability of the findings.

The researcher recommends that multiple sites or contexts should be used to confirm and further develop the theory. The researcher also recommends that the theoretical model could be used as a basis for understanding possible behaviour during and after disasters. The research result can be applied, as it can be applied in another context, but the audience of the research study are to make judgements of the transferability.

In this study, the modelling of the problematic behaviour was qualitative as the intention was to identify the mechanisms involved in understanding CR as perceived by relevant stakeholders. The SD models can be quantitative by defining measures and collecting appropriate data to allow to quantitative models. The researcher would recommend this as a further course of study.

## Conclusion

This research study proposes that communities in informal settlements are affected by disasters and the risks are high. Therefore, people need to know and understand CR to be able to build it within themselves or their communities, in the context of withstanding DR (Cutter et al. 2008; Lew et al. 2016).

One of the world’s most advanced DR policy frameworks for DRR may be found in South Africa, but implementing them happens to be a huge challenge because DR requires an essential developmental change (SA National Disaster Management Framework 2005). However, this study has indicated that DRR is a challenge and must be a priority to everyone. Although the focus of the study is on understanding CR in Phola Park context, it is likely that the challenges faced by this community are also faced by other rapidly growing large communities around South Africa. Therefore, the result or theoretical model in this study has further given standard mechanisms that could assist communities, sectors of government and other contexts to understand CR towards DRR.

The result of this article showed that the nine variables and the variable ‘public education and awareness’ are important ways for understanding and improving the level of understanding of CR in the vulnerable community in South Africa. The study also gives an insight into the potentials of the Phola Park community towards being a resilient community. However, the community still has some valuable understanding or knowledge that still needs proper guidance and help from external bodies and the government sector.

The participatory approach used for DRR management has assisted in understanding the viewpoints of the Phola Park community members towards understanding and building resilience and providing strategies that can be used to reduce DR in the future.

Furthermore, regarding the use of Internet for data collection that could be biased when trying to validate the research credibility (Metzger, Flanagin & Zwarun 2003), the validity threat was tackled with a thorough investigation of the authentication of the data, the qualifications and achievements of the authors and the endorsement of the websites as being trustworthy from the origin. The data derived from the Internet (like the university’s database) can further be validated with the type of triangulation that portrays the dependability of the data from the existence of the data or ideas in several origins and authors (Thurmond 2001).

In line with the understanding of the CR model identified, the impact that it has on the nine variables and the variable ‘public education and awareness’ in the informal settlement, has been recognised. It is apparent that through the variables, an individual’s level of understanding of resilience is affected, which impacts the community’s level of developing CR.

Through the application of data credibility, data dependability and data confirmability of the research and its results, the trustworthiness of the research process was established. Ethical approval was also secured and keenly followed to avoid any harm to all participants involved.
